# Réactions immunoallergiques graves aux antibacillaires: à propos de 10 cas

**DOI:** 10.11604/pamj.2014.19.152.5225

**Published:** 2014-10-15

**Authors:** Sabah El Machichi Alami, Sanae Hammi, Jamal Eddine Bourkadi

**Affiliations:** 1 Hôpital Moulay Youssef, CHU, Rabat, Maroc

**Keywords:** Tuberculose, traitement antibacilaire, effet secondaire, réaction immunoallergique grave, Tuberculosis, antibacillary treatment, side effect, severe allergic reaction

## Abstract

L'hypersensibilité aux antituberculeux est l'un des effets secondaires imprévisibles qui apparait chez 4 à 5 % de la population exposée et s’élève à 25% chez les sujets VIH positifs. Dans notre étude parmi 39 patients ayant présenté des réactions immunoallergiques, 10 avaient des formes graves. Le délai moyen d'apparition des signes était de 23 jours. Les réactions immunoallergiques observées étaient 5 cas de toxidermie généralisée fébrile, un cas de Dress syndrome, un cas de neutropénie, un cas de pancitopénie et 2 cas de thrombopénie. Tous nos patients avaient bien évolué cliniquement et bactériologiquement après l'adoption d'un régime thérapeutique excluant le ou les médicaments incriminés. En pratique, si l'effet indésirable imputé à un antituberculeux est grave, il est impératif de l'arrêter, de traiter l'incident et d'associer une autre molécule chez certains cas. Notre étude a montré une fréquence significative des complications graves probablement sous-estimée, surtout dans les pays fortement touchés par l'infection HIV.

## Introduction

Les allergies médicamenteuses peuvent être définies comme des réactions pathologiques induites par une prise médicamenteuse liées à un mécanisme immunologique [1]. L'hypersensibilité aux antituberculeux est l'un des effets secondaires imprévisibles qui apparait chez 4 à 5% de la population exposée et s’élève à 25% chez les sujets VIH positifs [1, 2].

## Méthodes

Etude rétrospective menée à l'hôpital Moulay Youssef de Rabat s’étalant sur une période de 18 mois (2013-2014). L'objectif était de recenser les patients ayant présenté des réactions immunoallergiques graves aux antibacillaires au cours de leur hospitalisation.

## Résultats

Parmi 39 patients ayant présenté des réactions immunoallergiques, 10 avaient des formes graves (25.6%). L’âge moyen de nos patients était de 37,5 ans avec un sexe ratio de 1. Le délai moyen d'apparition des signes était de 23 jours. Les réactions immunoallergiques observées étaient 5 cas de toxidermie généralisée, un cas de Dress syndrome, un cas de neutropénie, un cas de pancitopénie et 2 cas de thrombopénie. Le délai moyen de disparition des manifestations immunoallergiques était de 14 jours. Les caractéristiques des 10 patients et les médicaments réintroduits du moins au plus incriminé selon le tableau clinique sont résumés dans le Tableau 1. L'identification de l'antibacillaire responsable était faite par un test de réintroduction en commençant par le médicament le moins incriminé selon les tableaux cliniques. L'imputabilité de la pyrazinamide était retenue dans 5 cas, alors que celle de l’éthambutol, l'izoniaside, la streptomycine, la rifampicine et l'association pyrazinamide + éthambutole + isoniazide était retenue dans 1 cas respectivement. Le protocole de réintroduction est résumé dans le Tableau 2. Tous les patients avaient bien évolué cliniquement et bactériologiquement après l'adoption d'un régime thérapeutique excluant le ou les médicaments incriminés.

**Tableau 1:** Caractéristique des patients ayant nécessité l'arrêt définitif du médicament incriminé

Sexe	Age	Forme/Type	Délai (J)	TTT	Réaction	CAT	Résultat	Régime de sortie	
			A	D					
H	57	Mal de pott N.cas	45	21	2RHZE/4RH	Dress syndrome	arrêt ttt + CTC réintroduction progressive	réaction : H/Z/E Arrêt définitif : H/Z/E	SROEa
F	36	Pleurésie N.cas	10	7	2RHZE/4RH	éruption cutanée prurigineux des membres thrombopénie syndrome pseudo-grippal douleur abdominale avec vomissement	arrêt ttt + vit B6+antiémitique et anti H2 réintroduction progressive E- > Z- > H- > R	réaction : Z Arrêt définitif : Z	RHEO
F	36	Tuberculome cérébraux N.cas	45	21	2 SRHZE/7RH	toxidermie généralisée fièvre vomissement	arrêt ttt+ CTC réintroduction H- > R- > Z- > E- > S	réaction : E Arrêt définitif : E	RHZEa
H	12	Ganglionnaire N.cas HIV +	15	10	2RHZ/4RH	Syndrome pseudo-grippal neutropénie	Arrêt ttt + CTC + Anti H1 Réintroduction progressive Z- > H- > R	réaction : Z Arrêt définitif Z	RHE
F	33	Ganglionnaire N.cas	11	7	2RHZE/4RH	toxidermie généralisée fièvre	Arrêt ttt + CTC +anti H1 réintroduction H- > R- > Z- > E- > S	réaction: Z Arrêt définitif: z	RHE
H	51	Myélite N.cas	15	21	2SRHZE/4RH	toxidermie généralisée fièvre	Arrêt ttt + CTC +anti H 1 réintroduction H- > R- > Z- > E- > S	réaction: S Arrêt définitif: S	RHZE
F	14	TPM +	10	20	ERIPK4	toxidermie généralisée	Arrêt ttt + CTC +anti H 1 réintroduction E- > -I- > Z> R	réaction: Z Arrêt définitif: Z	RHE
F	23	Pleurésie	5	13	ERIPK4	Pancitopénie	Arrêt ttt + CTC +transfusion réintroduction E- > I- > R - > P	réaction: R Arrêt définitif: R	HZE
H	45	Tuberculose mutifocale HIV +	12	14	ERIPK4	Toxidermie généralisée	Arrêt ttt + CTC +anti H 1 réintroduction E- > P- > I- > R	réaction:H Arrêt définitif:H	ERZ
H	35	Miliaire HIV +	9	24	ERIPK4	thrombopénie	arrêt ttt + vit B6+antiémitique et anti H2 réintroduction progressive E- > Z- > H- > R	réaction : Z Arrêt définitif : Z	RHEO

N.cas : Nouveaux cas; ttt : traitement; CAT : conduite à tenir; A : délai d'apparition en jour; D : délai de disparition en jour; H : homme; F : femme; Ea : éthionamide; CTC : corticoïdes; O : oflocet; Vit B6 : vitamine B6.

**Tableau 2:** Protocole de réintroduction des médicaments dans notre série

	J1	J2	J3
H	50mg	150mg	Pleine dose
R	150mg	300mg	Pleine dose
Z	500mg	1g	Pleine dose
E	250mg	500mg	Pleine dose
S	250mg	500mg	Pleine dose

## Discussion

### Mécanismes des réactions immunoallergiques aux antibacillaires

Les antituberculeux peuvent induire des réactions d'hypersensibilité de type I à IV, selon la classification de Gell et Coombs [3, 4]: type I: anaphylaxie ou encore hypersensibilité immédiate; type II: hypersensibilité cytotoxique; type III: hypersensibilité semi retardée; type IV: hypersensibilité retardée. Les mécanismes en cause sont variés et loin d’être parfaitement élucidés. Lorsqu'elles sont authentiquement liées à une allergie, les réactions immédiates (dans l'heure qui suit la dernière prise) ou très accélérée, de type urticaire/angioedème ou choc par exemple sont le plus souvent IgE dépendantes. Quant aux réactions non immédiates, (exemple les exanthèmes maculo-papuleux), elles impliquent une activation des lymphocytes T spécifiques et répondent à plusieurs mécanismes immunologiques [5, 6] (Tableau 3).

**Tableau 3:** Classification des réactions immunologiques provoquées par les médicaments

Classification de Gell et Coombs	Type de réponse immunitaire	Caractéristiques physiopathologiques	Signes cliniques	Délai habituel d'apparition des symptômes
Type I	IgE	Activation des mastocytes et des basophiles	Choc anaphylactique Œdème de Quincke Urticaire Bronchospasme	De quelques minutes à 1 heure après la dernière prise médicamenteuse
Type II	IgG et FcR1	Cytotoxicité dépendant du FcR1	Cytopénie	5 à 15 jours
Type III	IgG ou IgM et Complément ou FcR1	Dépôts d'immuns complexes	Maladie sérique Urticaires Vascularites Lupus induit	7-8 jours pour la maladie sérique 7-21 jours pour les vascularites
Type Iva	Th1 (IFNγ)	Activation des monocytes	Eczémas	5-21 jours
Type IVb	Th2 (IL-4 et IL-5)	Inflammation éosinophilique	Exanthèmes maculopapuleux et bulleux	2-6 semaines pour le syndrome d'hypersensibilité médicamenteuse (DRESS) 2
Type IVc	Lymphocyte cytotoxique (perforine, granzyme B, FasL)	Lyse des kératinocytes médiée par les lymphocytes T CD4- ou CD8-	Exanthèmes maculopapuleux, bulleux et pustuleux	Moins de 2 jours pour l’érythème pigmenté fixe 7-21 jours pour les syndromes de Stevens- Johnson et de Lyell
Type IVd	Lymph T (IL-8/CXCL8)	Recrutement et activation des neutrophiles	Pustulose exanthématique aiguë généralisée	Moins de 2 jours

1FcR: récepteur membranaire cellulaire pour les immunoglobulines

2 DRESS: Drug Reaction with Eosinophilia and Systemic Symptoms

**Figure 1:**
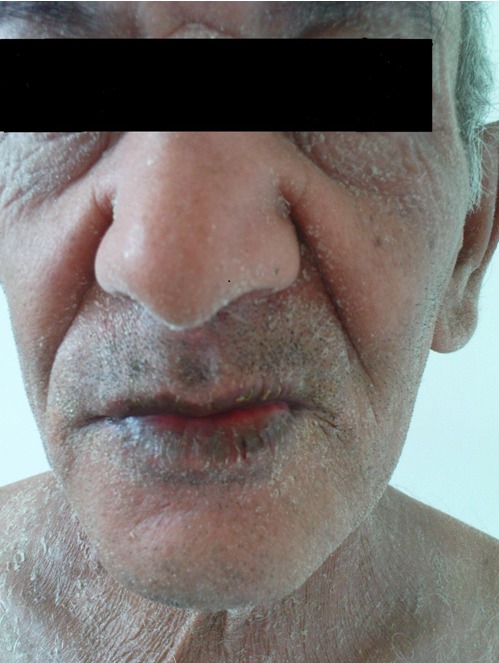
Exanthème maculeux du visage chez un patient qui a prsenté un DRESS syndrome attribué à 3 antibacillaires : isoniazide, pyrazinamide et éthambutol

**Figure 2:**
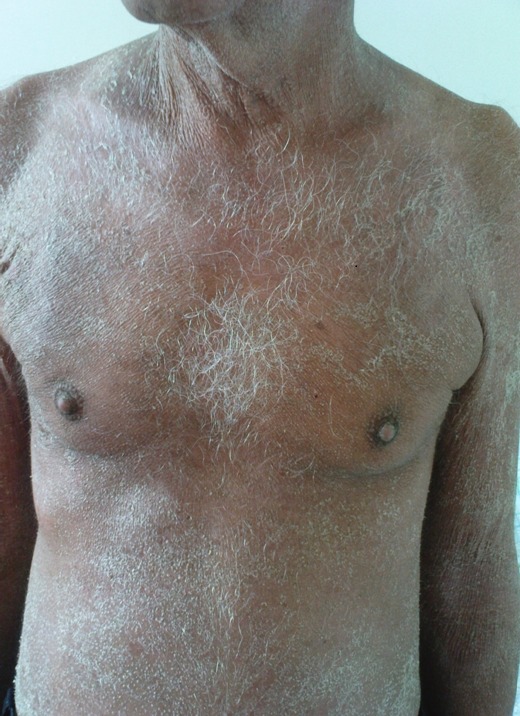
Exanthème diffus du tronc chez un patient qui a prsenté un DRESS syndrome attribué à 3 antibacillaires : isoniazide, pyrazinamide et éthambutol

**Figure 3:**
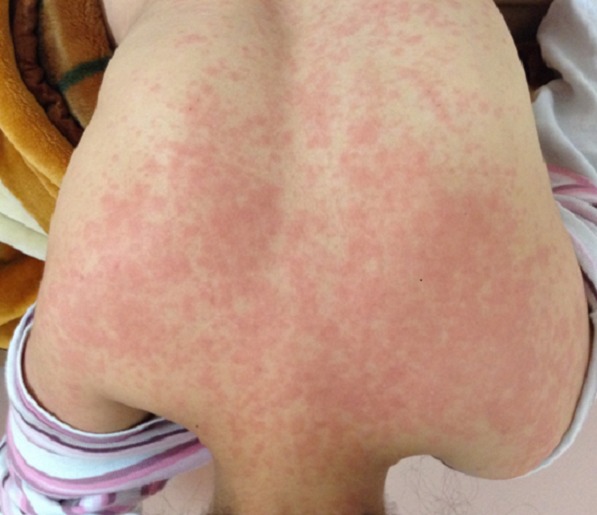
Toxidermie généralisée attribuée à la pyrazinamide

Les signes cliniques des réactions immunoallergiques aux antibacillaires: Les **manifestations cutanées** de l'allergie aux antibacillaires sont multiples, allant d'une simple urticaire au décollement cutané, parfois mortel. Des dermatoses sévères peuvent être observées avec les antituberculeux, comme le syndrome de Stevens-Johnson (SSJ) et de Lyell (SL). Bien qu'il soit exceptionnel, ces derniers ont été décrits avec la rifampicine et la streptomycine [7]. Ils surviennent 7 à 21 jours après le début du traitement. Pour Roujeau et Stern le risque est maximal pendant les deux premiers mois [8, 9]. Dans notre série le délai moyen des dermatoses sévères est estimé à 23 jours. Drira et al. [10] ont rapporté un cas de toxidermie à tous les antituberculeux. Dans une autre étude, deux cas de toxidermie généralisée sévère ont été notés, le premier imputé à la rifampicine [11] et le second à la streptomycine et au pyrazinamide [12]. Dans notre étude on rapporte un cas de Dress syndrome secondaire à 3 anti-bacillaires : isoniazide, pyrazinamide et éthambutol (Figure 1, Figure 2), et cinq cas de toxidermie généralisée secondaire à la streptomycine, à l’éthambutol,à l'isoniazide et au pyrazinamide (Figure 3). Ces cas sont rares mais graves pouvant mettre en jeu le pronostic vital, d'où la décision d'un arrêt définitif des médicaments incriminés.

**Les effets indésirables immunoallergiques hématologiques** sont représentés en premier par la **thrombopénie** qui est un accident rare mais grave qui peut être observé avec tous les antituberculeux. L'antibacillaire le plus fréquemment incriminé est la rifampicine, dans une moindre mesure l'isoniazide et de manière exceptionnelle le pyrazinamide [13, 14] La fréquence des**neutropénies** induites par le traitement antituberculeux est difficile à évaluer. Elle varie de 0,06% à 2,3% [15, 16] et est fréquemment associée à une thrombopénie [16]. Dans notre série on a décrit deux cas de thrombopénie et un cas de leucopénie secondaire à la pyrazinamide et deux autre cas de thrombopénie et pancitopénie dues à la rifampicine.

### Conduite à tenir devant les réactions immuno-allergiques aux antibacillaires

Notre conduite dépendait du type de l'allergie, de la sévérité du tableau clinique et du médicament suspecté. La première étape du diagnostic d'une allergie aux antibacillaires repose sur un interrogatoire minutieux afin de préciser: Le mode de début; La symptomatologie clinique: voir les personnes en aigu ou avoir des photos des lésions est important; La chronologie des symptômes: contacts antérieurs avec le médicament en cause, délai d'apparition après la dernière prise, effet de l'arrêt qui n'est pas toujours concluant...; Les antécédents du patient: notion d'incidents allergiques antérieurs, en présence ou en dehors de toute prise médicamenteuse, pathologies associées, prise médicamenteuse concomitante...; Les signes de gravité dont la présence doit faire suspecter, rechercher et traiter rapidement un choc anaphylactique, un œdème laryngé, un syndrome de Lyell ou de Stevens-Johnson, une vascularite, ou un syndrome d'hypersensibilité avec atteintes multi-organes ou DRESS. La présence de ces signes de gravité doit faire doser quelques paramètres biologiques (Tableau 4) et arrêter immédiatement le traitement [17].

**Tableau 4:** Signes de gravité (cliniques et biologiques) à rechercher rapidement devant toute suspicion d'allergie médicamenteuse

Signes d'alerte		Rechercher		Rapidement
		Signes		Diagnostics
Prurit palmoplantaire Chute tentionnelle	→	Autres signes d'anaphylaxie (urticaire/angioedème,TA)	→	Choc anaphylactique
	↗		↘	
Dysphonie, Hypersialorrhée				Œdème laryngé
Décollement cutané, bulles, signe de Nikolski, éruption douloureuse, érosions muqueuses	→	Bilan hydroéléctrolytique, NFS, TGO-TGP Complications systémiques	→	Syndrome de Lyell et Stevens-Johnson
	
Fièvre >40° EMP très étendue^1^ Infiltration du visage Polyadénopathies	→	NFS (éosinophiles) TGO-TGP,créatininémie, protiénurie	→	DRESS^2^
		
		
Purpura infiltre Nécrose cutanée	→	NFS (plaquettes) Compléments Créatininémie, protiénurie	→	vascularite

			

1 Examthème maculo-papuleux étendu à plus de 60% de la surface corporelle.

2 Drug reaction with eosinophilia and systemic symptoms ou syndrome d'hypersensibilité

Devant une forte suspicion clinique et en l'absence de moyen de réalisation des tests cutanés -qui ne sont pas encore validés pour les antibacillaires et aussi vu l'indisponibilité dans notre contexte de leur forme injectable - l'arrêt d'un ou de plusieurs médicaments associé à un traitement symptomatique en cas de nécessité (corticoïdes et / antihistaminiques’) est préconisée comme première étape. Par la suite (et seulement si nécessité absolue du médicament) on tente de réadministrer les antibacillaires à des doses progressives, l'un après l'autre en gardant en dernier le plus suspect de la réaction d'hypersensibilité, c'est **le test de réintroduction**. Ce test permettra d'identifier le médicament responsable. Alors devant les réactions allergiques graves, chez des patients ayant des formes pauci-bacillaires non étendues, **l'arrêt définitif** du médicament fortement suspecté est conseillé.

## Conclusion

Les effets indésirables des antituberculeux sont variables, parfois imprévisibles et potentiellement graves. Notre étude a montré une fréquence significative des complications graves qui restent sous-estimées (25.6% selon notre étude). La survenue d'un effet indésirable évoquant le rôle des antituberculeux pose le problème de l'identification du produit responsable en absence de validité du bilan allergologique clairement établie pour l'allergie aux antibacillaires
